# TRIM37 orchestrates renal cell carcinoma progression via histone H2A ubiquitination-dependent manner

**DOI:** 10.1186/s13046-021-01980-0

**Published:** 2021-06-15

**Authors:** Chenkui Miao, Chao Liang, Pu Li, Bianjiang Liu, Chao Qin, Han Yuan, Yiyang Liu, Jundong Zhu, Yankang Cui, Aiming Xu, Shangqian Wang, Shifeng Su, Jie Li, Pengfei Shao, Zengjun Wang

**Affiliations:** 1 Department of Urology, The First Affiliated Hospital of Nanjing Medical University, Nanjing, 210029 China; 2 Center for Quantitative Medicine, Duke-NUS Medical School, National University of Singapore, Singapore, SG 169857 Singapore; 3 Department of Urology, The First People’s Hospital of Changzhou, Changzhou, 213003 China

**Keywords:** Renal cell carcinoma, TRIM37, H2A ubiquitination, TGF-β1 signaling, Progression

## Abstract

**Background:**

Ubiquitylation modification is one of the multiple post-transcriptional process to regulate cellular physiology, including cell signaling, cycle regulation, DNA repair and transcriptional regulation. Members of TRIM family proteins could be defined as E3 ubiquitin ligases as they contain a RING-finger domain, and alterations of TRIM proteins are involved into a broad range of diverse disorders including cancer. TRIM37 is a novel discovered E3 ubiquitin ligase and acts as a oncoprotein in multiple human neoplasms, however its biological role in RCC still remains elusive.

**Methods:**

RCC microarray chips and public datasets were screened to identify novel TRIMs member as TRIM37, which was dysregulated in RCC. Gain or loss of functional cancer cell models were constructed, and in vitro and in vivo assays were performed to elucidate its tumorigenic phenotypes. Interactive network analyses were utilized to define intrinsic mechanism.

**Results:**

We identified TRIM37 was upregulated in RCC tumors, and its aberrant function predicted aggressive neoplastic phenotypes, poorer survival endings. TRIM37 promoted RCC cells EMT and malignant progression via TGF-β1 signaling activation, as a consequence of directly mediated by ubiquitinating-H2A modifications.

**Conclusions:**

Our findings identified a previously unappreciated role of TRIM37 in RCC progression and prognostic prediction. Importantly, we declared a novel ubiquitination-dependent link between TRIM ubiquitin ligases and TGF-β1 signaling in regulating cancerous malignancies.

**Supplementary Information:**

The online version contains supplementary material available at 10.1186/s13046-021-01980-0.

## Background

Kidney cancer is one of the ten leading cancer types and accompanies with approximately 85,680 estimated new cases in the United States according to cancer statistics 2020 [1]. Among which, renal cell carcinoma (RCC) is the most commonly cancer subtype [2]. Even though an accumulating detection of small renal lesions is observed through improved diagnostic strategy, around 1/3 of localized RCC will eventually develop local infiltration of distant metastases [3]. At this fatal stage, tyrosine kinase inhibitors (TKI) targeting vascular endothelial growth factor (VEGF) or mammalian target of rapamycin (mTOR) pathway arose and have been approved for the treatment of metastatic RCC [4, 5]. Although TKIs agents have shown robust clinical efficacy and prolonged the outcome of metastatic RCC, however inherent or acquired resistance are unavoidable at the end [6, 7]. Therefore, identification of novel and effective biomarkers to better predict RCC progression and ultimately cure RCC are higher clinical priority.

Tripartite motifs (TRIMs) are members which display E3 ubiquitin ligase activity as containing a common RING domain at the N-terminus [8]. TRIMs have been reported to participate various cellular processes including intracellular signaling, autophagy, transcription, carcinogenesis and cancer therapy [9]. Accumulating evidence has shown that TRIM proteins regulated carcinogenesis, along with disparate roles due to enormous family and different cancer subtypes. Some members like TRIM24, TRIM25, TRIM28 and TRIM29 act as oncogenic regulators in multiple cancers, whereas others like TRIM3, TRIM16 were reported to exert tumor-suppressive regulation [9].

In this study, we performed RCC microarrays chip analysis and identified one of TRIM family protein TRIM37 as an aberrant regulator in renal cancer. As a novel cancerous gene, TRIM37 could mono-ubiquitinate histone H2A to promote breast cancers tumorigenesis [10], and also regulate chemotherapeutic sensitivity via DNA repair way [11]. Furthermore, TRIM37 was also reported to dysregulate in multiple human cancer such as glioma [12, 13], gastric cancer [14], colorectal cancer [15, 16], lung cancer [17, 18], and hepatocellular carcinoma [19–21], and served as a oncoprotein via several classic signals including Wnt/β-catenin, MAPK/ERK, PI3K/Akt, NF-κB as well as epithelial-mesenchymal transition (EMT) process. However, the clinical significance and intrinsic mechanism of TRIM37 in RCC are completely unelucidated.

Our study illustrated that abnormal TRIM37 was correlated with adverse histological tumor grade, overall and relapse-free survival of RCC patients. Subsequent biological exploration confirmed TRIM37 was required for maintaining tumor cells migration and invasion phenotype in vitro and in vivo. Mechanically, TRIM37 directly interacted with histone H2A via ubiquitinated modification, sequentially activating TGF-β1/Smad2/3 signaling through the release of transcriptional regression. Taken together, our findings uncover that TRIM37 serves as promising biomarker for RCC progression and proposes a potential therapeutic target for cancer intervention.

## Materials and methods

### Clinical specimens and RCC microarrays

Matched RCC samples and adjacent non-tumorous specimens were obtained by radical or partially nephrectomy, samples of metastatic sites were obtained by CT-guided biopsy or local surgical excision at the First Affiliated Hospital of Nanjing Medical University. All diagnosis was confirmed by histopathological examination. The specimens were snap frozen in liquid nitrogen after surgery and stored at − 80 °C for use. RCC tissue microarray (TMA) contained cores from 133 RCC patients with known follow-up information and obtained from our hospital as described previously [22]. Informed consents of all patients were acquired in the study. The study design and protocol were approved by the ethic committee of hospital.

### Genomic, clinical and interactive network analysis

To study the genomic alteration status and the association between TRIM37, TGF-β1 signaling genes and the prognosis of RCC, gene altered condition, RNA-seq gene expression and following-up data were obtained from TCGA cohort through cBioPortal (https://www.cbioportal.org/) and Oncomine (https://www.oncomine.org/resource/login.html). TRIM37-ubH2A Chip-chip data was obtained from GEO database (https://www.ncbi.nlm.nih.gov/geo/). Protein-protein interaction network was performed by STRING database (https://string-db.org/), functional domain analysis was constructed by using Pfam (https://pfam.xfam.org/), InterPro (https://www.ebi.ac.uk/interpro/) and SMART (http://smart.embl-heidelberg.de/) databases. Enrichments of genes connection network were performed by GO and KEGG pathway analysis.

### Cell lines and culture

The human RCC lines (Caki-1, Caki-2, 786-O, ACHN) were obtained from the Cell Bank of Shanghai Institute of Cell Biology (Chinese Academy of Medical Science, Shanghai, China). The immortalized proximal tubule epithelial cell line (HK-2) was regarded as normal cells. Caki-1 and Caki-2 cells were maintained in McCoy’s 5A (Gibco, USA), 786-O and 769-P in RPMI-1640 (Gibco, USA), and HK-2, ACHN were propagated in DMEM (Gibco, USA) supplemented with 10% fetal bovine serum (Gibco, USA) and 1% penicillin (Invitrogen) respectively. All cells were developed at 37 °C in an incubator with 5% CO_2_.

### Antibodies and reagents

Primary antibodies were obtained from: rabbit anti-TRIM37 (Abcam), mouse anti-TRIM37 (Millipore Sigma), rabbit anti-histone H2A (Cell Signaling Technology), rabbit anti-Ubiquityl-Histone H2A (Cell Signaling Technology). Anti-TGF-β1, Smad2/3, Phospho-Smad2, Phospho-Smad3, ZEB-1, Snail, Claudin-1, N-cadherin, Vimentin and GAPDH were obtained from Cell Signaling Technology. Secondary antibodies including anti-mouse IgG, HRP-linked, anti-rabbit IgG, HRP-linked were gained from ZSGB-BIO. Alexa Fluor Secondary antibody (Spectrum 488, 594) were obtained from Beyotime Biotechnology. PRT4165 was obtained from Selleck chem,

### Recombinant human TGF-β1 stimulation

Recombinant Human TGF-β1 (Rh TGF-β1) (R&D system, USA) was reconstituted at 20 μg/mL in sterile 4 mM HCl containing 0.1% bovine serum albumin for use. Cells with sh-TRIM37 were seeded in 6-well plates and grew at 50% confluence before stimulation. The Rh TGF-β1 stimulation was performed at concentration of 2 ng/mL for 2 days before cells were harvested for further experiments.

### Plasmid and shRNA transfection

The lentiviral vectors containing small hairpin RNA (shRNA) targeting TRIM37 and negative control (NC) were obtained from Genechem (Shanghai, China). The shRNA target sequences were listed as the following: shRNA-Hu6-MCS-Ubiquitin-EGFP-IRES-puromycin (shTRIM37–1: 5′-GCTGAAGAATAAGCTTATA-3′, shTRIM37–2: 5′-GCTACGAGAACTAGTAAAT-3′). Lentiviral transduction was performed in Caki-1, Caki-2 cell lines. Pools of stable transduction were selected with puromycin (3 μg/ml) for 2 weeks. Overexpression of TRIM37 with His-tagged was constructed into PDS085_pL-MCS plasmid vector for lentiviral package (Novobio, Shanghai, China). Lentiviral transduction was performed in Caki-1, 786-O and ACHN cell lines. Pools of stable transductants were generated by selection using blasticidin (4 μg/ml) for 2 weeks.

### RNA extraction, qRT-PCR and ChIP assay

Total RNA was drawn from cell lines and tissues by using TRIzol reagent (Invitrogen, Carlsbad, CA, USA). Complementary DNA (cDNA) was synthesized by using PrimeScript RT Master Mix (TaKaRa, Kyoto, Japan). Reverse transcription polymerase chain reaction (RTPCR) was conducted by using HiScript II (Vazyme, Shanghai, China) in accordance with the manufacturer’s guidelines. Quantitative RT-PCR (qRT-PCR) was performed using SYBR Green I (Vazyme, Shanghai, China) on ABI 7900 system (Applied Biosystems, Carlsbad, CA, USA). β-actin was employed as a normalization. For PCR, we used the following primers (Realgene, Nanjing, China): TRIM37 (5′-ATGAGCGTGTTCGGGAAATTAG-3′ and 5′-TCACTCTTACTACAAGACCGCAA-3′); β-actin (5′-ACTGGAACGGTGAAGGTGAC-3′ and 5′-AGAGAAGTGGGGTGGCTTTT-3′).

The Chromatin Immunoprecipitation Kit (Catalog # 17–371, Millipore) was used to perform ChIP assays, in line with the manufacturer’s protocols. Briefly, cells with stable lentiviral transduction were selected and purified for subsequent antibodies immunoprecipitation, antibodies against TRIM37, H2A and negative control IgG as described above were used in the ChIP assays. ChIP DNA products were amplified with specific primer of TGF-β1 promoter (5′-GGCAGTTGGCGAGAACAGT-3′ and 5′-CTGGGGTCAGCTCTGACAGT-3′).

### Cell migration, invasion and scratch wound assay

The migratory and invasive abilities of RCC cells were measured using transwell assays and wound-healing assays, respectively. For transwell assays, 3 × 10^4^ cells were seed into upper chambers in serum-free medium, with or without Matrigel, and medium with 20% FBS was added into the lower chamber for subsequent observations. After incubation for 48 h, the cells remaining in the upper chamber were wiped off, and the cells had migrated to the bottom surface were fixed with 4% paraformaldehyde and stained with crystal violet for image. For wound-healing assay, cells were seeded into 6-well plate for 24 h, then a wound was created using a sterile 200 μL pipette tip. Cells were allowed to migrate and heal the wound, and the migration distance was monitored under a microscope.

### Three-dimensional (3D) tumor spheroid invasion assay

A density of 4 × 10^3^/200 μL RCC cells with well-established transduction were collected and seeded into ultra-low attachment (ULA) 96-hole round base plate and cultured for 4 days. Afterward, remove 100 μL of media from each well with pre-cold pipette tip, then 100 μL of Matrigel matrix (3 mg/ml, Corning) was added to the hole at the bottom of each well. The ULA 96-hole plate was then transferred to the incubator at 37 °C to solidify the Matrigel, and 1 h later 100 μL of media was added to each well. The tumor spheroid image was captured at 24-72 h for further comparison.

### Cell proliferation and viability assay

RCC cells with different pretreatment were plated in 96-well plates at a density of 1000cells in 100 μL growth media. At indicated time points (24, 48, 72, 96 h), cells were replaced with 90 μL fresh media and 10 μL Cell Counting Kit-8 (CCK-8) reagents. Absorbance at 450 nm of each well were read using a microplate reader (Tecan, Switzerland) to determine the cells viability.

### Two-dimensional (2D) colony formation and anchorage-independent soft agar growth

2D colony formation assays were performed by seeding 2 × 10^3^ of RCC cells into per well of 6-well plates. Cells were maintained in culture media for 2 weeks. After, cells were incubated with fixative solution and colonies were stained with crystal violet for image. To perform 3D soft agar assay, 5 × 10^3^ of 786-O cells were suspended in media with 0.6% Noble agar and placed onto bottom layers gel with media containing 1% Noble agar in 6-well plates. Afterwards, cells were maintained in a 37 °C incubator for 4 weeks. For every week, 200-μL of complete media were added onto the upper layer to prevent desiccation. Colonies were monitored and calculated under an optical microscope.

### Luciferase reporter assays

The promoter of TGF-β1 was constructed into pGL3-reporter plasmid for luciferase reporter assay. RCC cells with NC or shTRIM37 were transiently transfected with TGF-β1 luciferase reporter using X-tremeGENE transfection reagent (Sigma, USA). Luciferase activity was measured using a ONE-Glo luciferase assay system (Promega, USA) according to the manufacturer’s instructions.

### Xenograft tumor assay

All animal experiments were performed with approval of the Institutional Animal Care and Use Committee of Nanjing Medical University (IACUC-IACUC-1801013).

For subcutaneous xenograft models, 2 × 10^6^ of 786-O cells with stably overexpressing TRIM37 and negative control cells were pelleted and suspended in a total of 100 μL volume containing 50 μL PBS and 50 μL Matrigel matrix on ice, then cells were subcutaneously injected into 5-week-old male BALB/c nude mice (Model Animal Research Center of Nanjing University). The tumor growth was monitored every 5 days using a vernier caliper, and the tumor volume was calculated according to the formula: = length×width^2^/2. The mice were monitored for 30 days. At the end point, the mice were sacrificed, and the tumors were dissected and weighted. Tumor samples were used for further analysis. For metastatic xenograft models, 1 × 10^6^ of 786-O_Luciferase cells with previously established TRIM37 overexpression or wild type were suspended in 100 μL PBS solution and injected into 5-week-old male BALB/c nude mice through tail veins. Two weeks later, the bioluminescence density was monitored every week for 4 weeks. To produce the bioluminescence system, mice were intraperitoneally injected with D-luciferin sodium salt (150 mg/kg) and the luminescent signal were detected with an IVIS spectrum Xenogen Imaging system (Caliper Life Sciences) after 10 min. The mice were anaesthetized using 2% isoflurane. All mice were sacrificed at the end point, and their lungs were resected for subsequent studies.

### Immunoprecipitation (IP) and immunoblot assay

To examine endogenous TRIM37, H2A and TGF-β1 interaction, RCC cells were harvested and lysed in IP buffer (50 mM Tris-HCl, pH 8.0, 120 mM NaCl, 0.3% NP40, 1 mM EDTA, 1 x protease inhibitor, phosphatase inhibitors). Then the lysates were incubated with specific target or IgG antibody at 4 °C overnight with constant rotation. Afterward, Protein A/G magnetic beads (Thermo Fisher Scientific) were added for another 2 h to capture the immune complexes. Beads were then washed for 3 times with IP buffer, and immunoprecipitated protein were eluted with SDS loading buffer and analyzed by Western Blot.

Cells and tissues were lysed with RIPA lysis buffer (Beyotime Biotechnology, Shanghai, China). Proteins were extracted and then quantified using a Bicinchoninic acid (BCA) kit (Beyotime Biotechnology, Shanghai, China). For Western Blot procedure, proteins were loaded and separated on 10% SDS/PAGE gel, and then transferred onto polyvinylidene difluoride membranes (PVDF) (Millipore, Bedford, USA, ISEQ00010). The membranes were blocked in 5% non-fat milk and then incubated with specific primary antibodies at 4 °C overnight. After being incubated with HRP conjugated secondary antibodies, the blots were visualized using the ECL system (Bio-Rad).

### Immunofluorescence (IF) and immunohistochemistry (IHC) assay

Pretreated RCC cells were plated in 60 mm chamber dishes 24 h prior to experiment. Cells on coverslips were washed 2 times in PBS, then fixed with 4% paraformaldehyde for 20 min, and permeabilized with 0.5% Triton X-100 at room temperature (RT) for 10 min. Then, cells were washed 3 times in PBS and blocked with 2% BSA for 30 min before incubated with indicated primary and secondary antibodies at 4 °C overnight or at RT for 1 h, respectively. After, DAPI were added to stain the nucleus and slides were observed using a fluorescence microscope (Olympus).

IHC assays were performed as described previously [23]. Formalin-fixed, paraffin-embedded tissue slides were dewaxed with xylene and rehydrated by a graded series of alcohols, followed by antigen retrieval and block with 5% BSA for 60 min. Incubation was carried out at 4 °C for overnight with the primary antibody. The evaluation of protein staining was separately and independently conducted by two experienced pathologists without knowledge of clinical data. The IHC staining signals were detected using Amend Allred scoring system as previously described [24]. The intensity of staining were scored as previous describing approaches [25].

### Statistical analysis

All experiments were repeated independently with similar results at least three times. The statistical analysis was carried out with SPSS 25.0 software (SPSS Inc., Chicago, USA) and GraphPad Prism 8 software (GraphPad Software, USA). The student’s t-test and one-way ANOVA were performed to determine the statistically significance in comparison with different groups. The correlation of the expressions of TRIM37 and TGF-β1 was established by Pearson correlation coefficient and linear regression model. The survival analysis was constructed by using the Kaplan-Meier method and analyzed by the log-rank test. *P < 0.05, **P < 0.01, ***P < 0.001, ****P < 0.0001 was considered statistically significant in all tests, NS was regarded as not significant.

## Results

### TRIM37 is overexpressed and associated with poor outcome of RCC

**Fig. 1:**
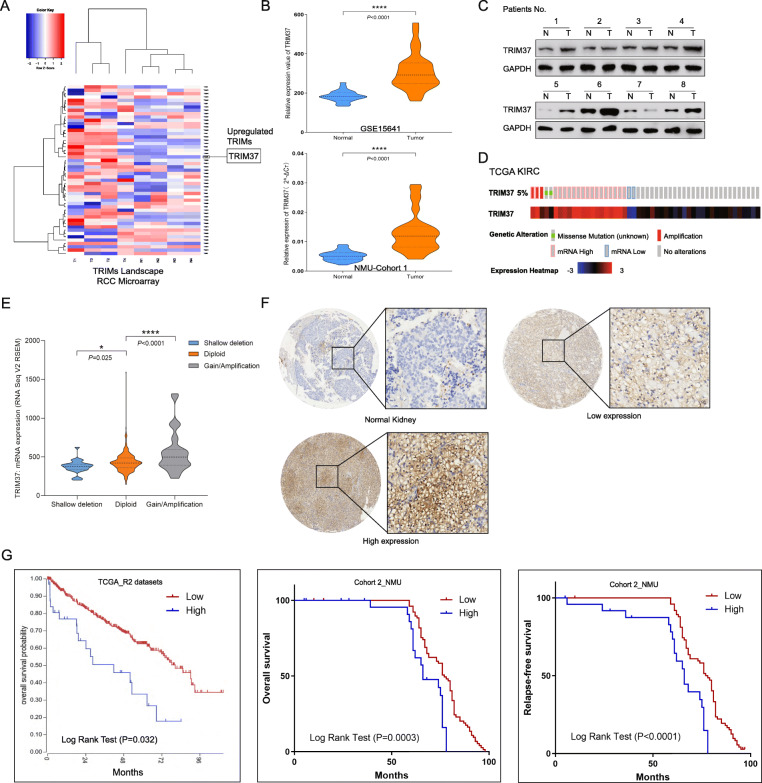
TRIM37 is overexpressed and associated with poor outcome of RCC. **A,** The heat map of microarray chip in RCC tumors showed that TRIM37 was upregulated in tumor specimens than normal tissues (Foldchange> 1.5, *P* < 0.05 was regarded as significant)**. B,** GEO datasets and NMU_RCC cohort1 validated abnormal TRIM37 expression in tumors than normal tissues. **C,** TRIM37 protein levels was elevated in tumorous samples than normal ones of RCC patients. **D,** TCGA datasets showed 5% alteration of TRIM37 in RCC patients, 69% of which were mRNA high. **E**, RCC tumors with gain or amplification of TRIM37 had significantly higher transcription levels than shadow deletion or diploid alterations in TCGA cohort. **F,** IHC staining showed TRI37 intensity was higher than those in normal kidney tissues. **G,** Kaplan-Meier survival analysis declared TRIM37-high patients had poorer cumulative overall and relapse-free survival in NMU_RCC cohort 2. High TRIM37 also predicted shorter overall survival time in TCGA RCC patients

RCC microarray chip was used to assess the expression of TRIMs protein (Fig. 1A, Figure S1A). Among which, TRIM37 was most significantly upregulated in RCC specimens compared with para-cancerous tissues, and this observation was also confirmed by GEO datasets (GSE15641, Fig. 1B). In 47 of RCC samples from NMU_RCC cohort 1 (Table S1), TRIM37 mRNA level was statistically elevated in tumor tissues than normal tissues (Fig. 1B) and in Grade 3–4 (*P <* 0.0001) tumors, but not in Stage T3-T4 tumors (*P =* 0.0788) (Figure S1B). The protein of TRIM37 showed similar expression trend (Fig. 1C). Analysis of The Cancer Genome Atlas (TCGA) demonstrated that TRIM37 alterations exist in 5% of ccRCC cases, among which strikingly the majority of alterations are mRNA rises (16/23, 69%) (Fig. 1D). A 5% alteration rate of TRIM37 is comparable with those well-established ccRCC putative genes [26] such as PIK3CA (8%), MTOR (12%), PTEN (8%) and TP53 (9%) (Figure S1C). When stratified by TRIM37 copy number status, RCC tumors with gain or amplification of TRIM37 had significantly higher transcription levels than shadow deletion or diploid alterations (Fig. 1E). The schematic alterations of these genes in pRCC was also shown in Figure S1D-E.

To analyze the clinical characteristics of TRIM37 in RCC, we determined TRIM37 protein level in a set of RCC tissue microarrays (TMAs) containing survival information of 133 tissue cores from NMU_RCC cohort 2. Significantly upregulated TRIM37 score was observed in RCC tumors (Fig. 1F). Kaplan-Meier survival analysis declared that TRIM37-high patients experienced poorer cumulative overall survival (*P =* 0.0003) and relapse-free survival (*P* < 0.0001) than TRIM37-low patients (Fig. 1G). Analysis of survival status from TCGA ccRCC cohort revealed that TRIM37 upregulation predicted decreased overall survival benefit (*P =* 0.032, Fig. 1G). Assessment of clinic-pathological value in TMAs showed TRIM37 score was significantly associated with advanced histological tumor grade (*P* < 0.0001), patients’ survival (*P =* 0.0002) and recurrence (*P =* 0.035), but not in patients age (*P =* 0.209) and gender (*P* = 0.635), tumor size (*P* = 0.088), tumor histology (*P* = 0.96) and tumor stage (0.057) (Table 1 and S2). These data firstly uncover TRIM37 as an abnormal and predictive biomarker for higher tumor grade and poorer outcomes of RCC patients.

**Table 1:** Correlations between TRIM37 expression and clinicopathological factors

Characteristics	Number	TRIM37 expression		
		Low (*n* = 79)	High (*n* = 54)	*P* value
Age (year)				
<60	80	51	29	0.209
≥60	53	28	25	
Gender				
Male	83	48	35	0.635
Female	50	31	19	
Tumor size (cm)				0.088
≤4	71	47	24	
>4	62	32	30	
Histology				0.96
Clear cell RCC	118	70	48	
Others	15	9	6	
Histological grade				**5.25543E-05**
I-II	110	74	36	
III-IV	23	5	18	
Tumor stage				0.057
T1	115	72	43	
T2-T4	18	7	11	
Death				
Yes	18	3	15	**0.000205578**
No	115	76	39	
Recurrence				
Yes	12	4	8	**0.035**
No	110	71	39	

### TRIM37 is critical for the migration, invasion and EMT process of RCC cells

**Fig. 2:**
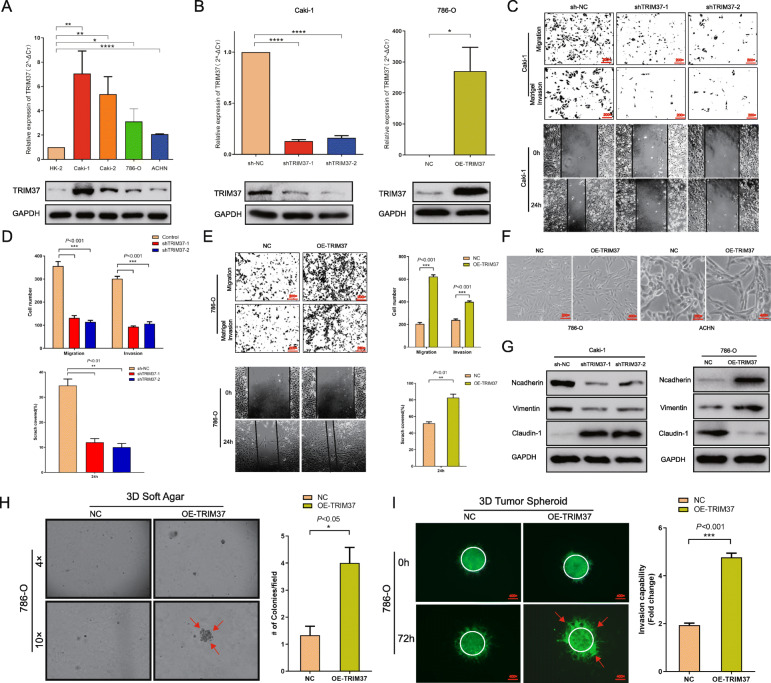
TRIM37 is critical for the migration, invasion and EMT process of RCC cells. **A,** Expression intensity of TRIM37 in RCC cell lines (Caki-1, Caki-2, 786-O, ACHN) was higher than normal kidney lines (HK-2). **B,** Validation of knocking down or overexpressing TRIM37 cell models (Caki-1, 786-O). **C-E,** Knockdown of TRIM37 attenuated cell migration and Matrigel invasion ability, overexpression of TRIM37 enhanced cell migration and Matrigel invasion ability. **F,** Overexpression of TRIM37 in RCC cells gradually exhibited a spindle-shaped and more elongated morphology. **G,** Knockdown of TRIM37 inhibited the expression of mesenchymal markers including N-cadherin, Vimentin and increased the expression of epithelial marker Claudin-1 in Caki-1 and 786-O lines. Overexpression of TRIM37 displayed adverse trends. **H,** Upregulating TRIM37 promoted 3D soft agar colony formative capability. **I,** Upregulating TRIM37 promoted 3D tumor spheroid invasive potential

We further aimed to ascertain the functional significance of TRIM37 in RCC. The human RCC lines (Caki-1, Caki-2, 786-O, ACHN) expressed higher TRIM37 compared to normal HK-2 cells (Fig. 2A). Performing functional assays, TRIM37 suppression notably attenuated migrative and invasive capabilities of Caki-1 and Caki-2 cells (Fig. 2B-D, S2B-C), while overexpression of TRIM37 enhanced the abilities in 786-O and ACHN cells (Fig. 2E, Figure S2D-E). Knocking down TRIM37 inhibited cells growing rates and colony formation ability in Caki-1 and Caki-2 cells (Figure S2F-G). However, overexpressing TRIM37 only showed slight increase to cell proliferative and clonogenic potentials in 786-O and ACHN cells (Figure S2H-I). These results indicated that TRIM37 might focus more on the migrative and invasive potential of RCC cells other than cell proliferation.

Meanwhile, we interestingly found that partial RCC cells with TRIM37 overexpression exhibited a spindle-shaped and more elongated morphology (Fig. 2F). RCC is known to originate from the primary renal tubular epithelial system and most of cell lines exhibit epithelial morphology [27]. The morphological change represented a loss of epithelial phenotypes and gain of mesenchymal phenotypes, which called EMT process (Figure S2J) [28, 29]. EMT is a crucially phenotypic plasticity process for migratory and invasive properties in cancer [30], blocking of TRIM37 significantly resulted the downregulation of mesenchymal cell markers of N-cadherin and Vimentin, as well as the upregulation of epithelial marker Claudin-1 (Fig. 2G, Figure S2K). Conversely, overexpressing TRIM37 showed reverse expression trend (Fig. 2G, Figure S2K). We then performed soft agar colony assay and found TRIM37 promoted 3D colony formative potential (Fig. 2H). Next, to determine the metastatic capability of RCC cells of 3D models, we conducted tumor spheroid invasion assay and showed that elevating TRIM37 significantly accelerated tumor spheroid invasive ability in RCC cells (Fig. 2I). In summary, TRIM37 could enhance RCC cells tumorigenic and invasive skills, and function as an oncogenic factor to promote tumor progression via EMT procedure.

### TRIM37 promotion of RCC progression and EMT relies on canonic TGF-β1/Smad2/3 signaling

**Fig. 3:**
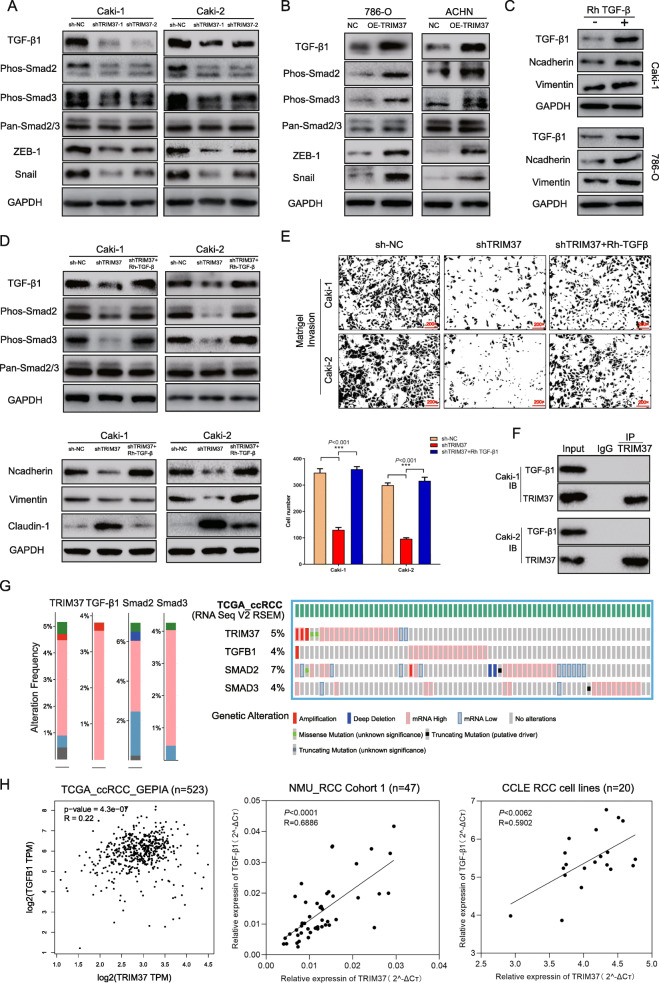
TRIM37 promotion of RCC progression and EMT depends on canonic TGF-β1/Smad signal. **A-B,** TRIM37 ablation inhibited TGF-β1, Smad2, Smad3, ZEB-1 and Snail activity in Caki-1 and Caki-2 cells, while overexpression enhanced their expression in 786-O and ACHN cells. **C,** Rh TGF-β1 stimulator reversed TGF-β1/Smad2/3 signaling inhibited by TRIM37 silencing. **D,** Rh TGF-β1 stimulator reversed EMT plasticity phenotypes (N-cadherin and Vimentin increased, Claudin-1 decreased) inhibited by TRIM37 silencing. **E,** TRIM37 knockdown inhibited Matrigel based invasion, and Rh TGF-β1 stimulator restored the invasive potential suppressed by TRIM37 downregulation in Caki-1 and Caki-2 cells. **F,** Immunoprecipitation assay using TRIM37 antibody indicated that TRIM37 could not directly interact with TGF-β1 in Caki-1 and Caki-2. **G,** Altered frequencies of TRIM37 and TGF-β1 signaling gene in TCGA datasets, including TRIM37 (5%), TGF-β1 (4%), Smad2 (7%) and Smad3 (4%). H, Correlative analysis declared that TRIM37 expression positively correlated with TGF-β1 expression in TCGA datasets, NMU_RCC cohort1 and CCLE RCC cell lines analysis. **I,** GSE11151 datasets showed TGF-β1 expression was upregulated in tumors than normal tissues. **J,** Overall survival role of TGF-β1 in TCGA datasets. High TGF-β1 expression predicted shorter overall survival time in TCGA RCC cohort

EMT pattern is usually driven by transcriptional factors like SNAIL, ZEB and TWIST [31, 32], and induced by aberrant TGF-β1 signaling [33–35]. First, we examined whether TGF-β1 signaling was affected by TRIM37. The results showed that TGF-β1 expression was downregulated after silencing TRIM37, whereas increased when TRIM37 was overexpressed (Fig. 3A, B). Moreover, TGF-β1 inducing phosphorylation-Smad2 (Phos-Smad2), phosphorylation-Smad3 (Phos-Smad3), related factors like Snail and ZEB1 levels also remarkably changed (Fig. 3A, B). Then, we observed that exogenous Rh TGF-β1 significantly increased N-cadherin and Vimentin expression in RCC cells, indicating that Rh TGF-β1 could promote EMT process (Fig. 3C). Phos-Smad2, Phos-Smad3 and EMT phenotypes were also reversed by Rh TGF-β1 stimulation in TRIM37 knockdown cells (Fig. 3D). Functional assays demonstrated that ectopic Rh TGF-β1 activity could signally rescue the invasive capability of RCC cells suppressed by TRIM37 downregulation (Fig. 3E). These findings indicated that TRIM37 might promote RCC metastatic potential and EMT process through TGF-β1/Smad2/3/Snail/ZEB1 signaling. To clarify whether TRIM37 could directly interact with TGF-β1, we next performed immunoprecipitation assays. Results showed that anti-TRIM37 failed to precipitate TGF-β1 protein (Fig. 3F), indicating its mediating TGF-β1 signaling might go through an indirect manner.

Then, we identified the alterations of TRIM37 and TGF-β1 signaling in TCGA datasets. The prominent alterations type of TRIM37 and TGF-β1 signaling were mRNA high (Fig. 3G, Figure S3A). Further correlative analysis demonstrated that TRIM37 level positively correlated with TGF-β1 expression in TCGA datasets (R = 0.22, *P* < 0.05), NMU_RCC cohort 1 (R = 0.6886, *P* < 0.05) and CCLE RCC lines (R = 0.5902, *P* < 0.05) (Fig. 3H). We next analyzed the clinical significance of TGF-β1 in RCC. GEO datasets (GSE11151) showed that TGF-β1 was significantly upregulated in RCC tissues (Figure S3B). High TGF-β1 level also predicted poorer overall survival of TCGA RCC patients, preliminarily indicating the prognostic and oncogenic role of TGF-β1 in RCC (Figure S3C).

Taken together, the promoting role of TRIM37 in RCC progression and EMT process is mainly dependent upon TGF-β1/Smad2/3 signal and activating Snail/ZEB1 transcriptional factors.

### TRIM37 activating TGF-β1 signaling in RCC progression requires histone H2A ubiquitinated modification

**Fig. 4:**
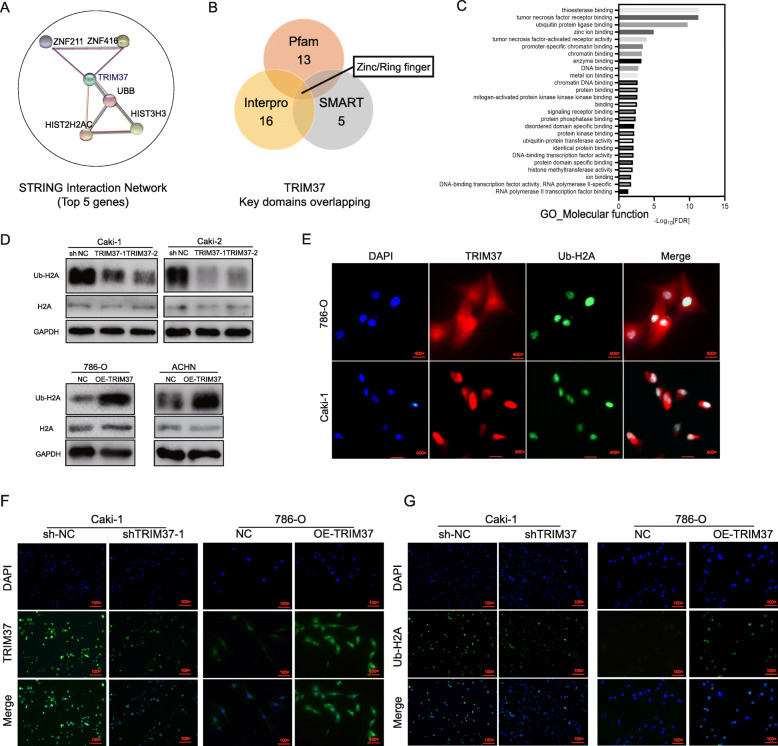
TRIM37 connected-signatures analysis and its promoting role in ubiquitinating histone H2A. **A,** Protein-protein interaction (PPI) network analysis of TRIM37 involving modules based on STRING database, the top 5 genes were HIST2H2AC, HIST3H3, UBB, ZNF211 and ZNF416. **B,** Functional domains analysis of TRIM37 using Pfam, InterPro and SMART databases, indicating Zinc/Ring finger as a crucial site for TRIM37 activity. **C,** GO molecular function analysis declared thioesterase binding, tumor necrosis factor receptor binding and ubiquitin protein ligase binding of TRIM37 connected genes. **D,** Knockdown of TRIM37 decreased ub-H2A levels of Caki-1 and Caki-2 cells, overexpression of TRIM37 enhanced conversely in 786-O and ACHN cells. **E,** Co-localized IF staining displayed the distribution of TRIM37 and ub-H2A: TRIM37 was primarily distributed in the whole cell while ub-H2A conservatively localized in cell nucleus. **F,** IF staining validation of knocking TRIM37 in Caki-1 cells and overexpression in 786-O cells. G, Restrained TRIM37 inhibited the expression intensity of ub-H2A in Caki-1 cells, whereas elevating TRIM37 put up stronger staining signals in 786-O cells

To provide insight into the underlying mechanism how TRIM37 interact with TGF-β1 signaling and EMT process, we performed protein-protein interaction (PPI) network analysis using STRING datasets. A total of 26 nodes were identified (Figure S4A), among which the top 5 genes were HIST2H2AC, HIST3H3, UBB, ZNF211 and ZNF416 (*P* < 1.0e-16, Fig. 4A). To deeply understand the functional motifs of TRIM37, we used Pfam, InterPro and SMART databases (Table S3), and observed that Zinc/Ring finger domain as a crucial site for the activity of TRIM37 (Fig. 4B). GO enrichments and KEGG pathway analysis of most connected genes indicated that TRIM37 dominantly displayed its function via ubiquitylating approaches (Fig. 4C, Figure S4B-D). Moreover, chip-chip analysis of GSE48196 identified a total of 7638 genes which were simultaneously bound by TRIM37 and enriched for ub-H2A (Figure S4E). Interestingly, TGF-β1 was also included in the overlapping lists of which TRIM37 and ubH2A co-enriched. Therefore, we considered the similar possibility that TRIM37 acted as a histone H2A ubiquitin ligase in RCC, thus promoted TGF-β1 signaling and RCC progression. Knocking down TRIM37 dramatically decreased the level of ubiquitination of H2A, and overexpression of TRIM37 remarkably augmented ub-H2A levels (Fig. 4D). Furthermore, we identified that TRIM37 was primarily distributed in the whole-cell chamber while ub-H2A conservatively localized in cell nucleus (Fig. 4E). Immunofluorescent staining proved that restrained TRIM37 inhibited the expression intensity of ub-H2A, whereas elevated TRIM37 put up stronger staining signals (Fig. 4F-G).

**Fig. 5:**
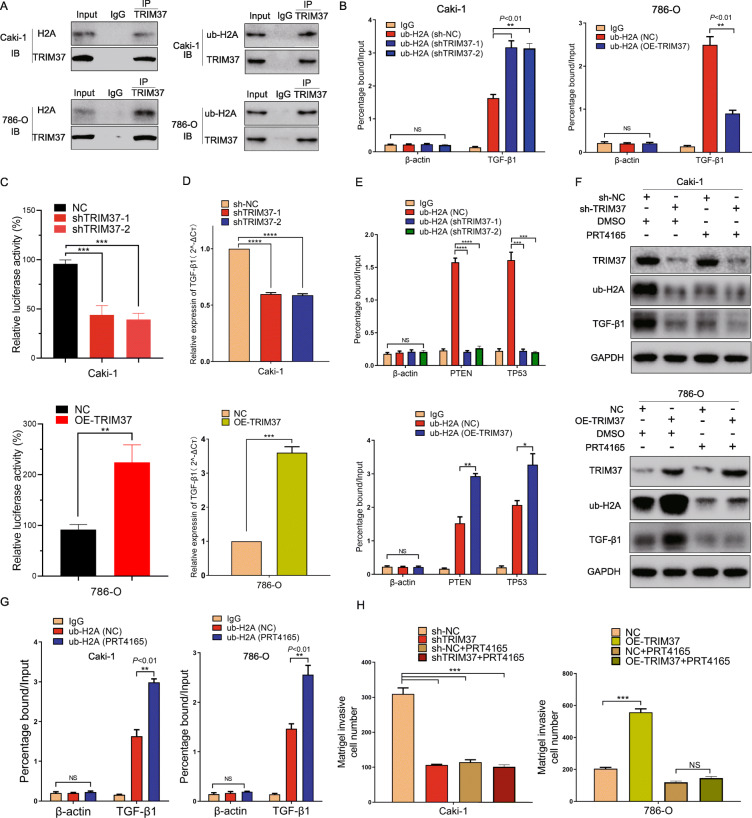
TRIM37 activating TGF-β1 signaling in RCC progression requires histone H2A ubiquitinated modification. **A,** TRIM37 directly interacted with histone H2A and ub-H2A in RCC cells with immunoprecipitation assay. **B,** Ablation of TRIM37 significantly improved ub-H2A levels at the TGF-β1 promoter site in Caki-1 cells, while overexpressing TRIM37 in contrast decreased ub-H2A enrichments in 786-O cells. **C,** knockdown of TRIM37 significantly reduced the luciferase activity of TGF-β1 of Caki-1 cells, overexpression of TRIM37 on the contrast enhanced its activity. **D,** TGF-β1 mRNA level decreased or elevated in knockdown or overexpressing TRIM37 models, respectively. **E,** TRIM37 knockdown significantly reduced ub-H2A occupancy at the promoter sites of PTEN and TP53 in Caki-1 cells, indicating the decreasing repression of transcription, while overexpressing TRIM37 showed opposite effects in 786-O cells. **F,** Specific ub-H2A inhibitor PRT4165 significantly abolished H2A ubiquitinating levels, TGF-β1 expression in sh-NC or sh-TRIM37 cell models, with TRIM37 expression no change, indicating that TRIM37 functioned at the upstream of both H2A and TGF-β1. In TRIM37 overexpression cells, PRT4165 could obviously inhibited ub-H2A and TGF-β1 expression, which were upregulated by TRIM37. **G,** PRT4165 could increase the occupancy of ub-H2A at TGF-β1 promoter site in Caki-1 and 786-O lines, which is similar to TRIM37 ablation. **H,** PRT5165 significantly restrained the invasive capabilities of cells with TRIM37 knockdown or overexpression, indicating the functional effects of ubiquitinating inhibition

To further verify the interaction between TRIM37 and histone H2A, we performed immunoprecipitation assay and found that TRIM37 could directly interact with histone H2A and ub-H2A in RCC cell lines (Fig. 5A). Reportedly, TGF-β1 was a co-bound factor for TRIM37 and ub-H2A, implying the occupancy of ub-H2A in TGF-β1 promoter might affect its transcription activity [10]. To confirm this possibility, we performed ChIP experiment to test ub-H2A levels at the TGF-β1 promoter site. Despite reducing ub-H2A expression levels, ablation of TRIM37 significantly improved ub-H2A levels at the TGF-β1 promoter site (percentage bound around 193% than NC cells), while overexpressing TRIM37 in contrast decreased ub-H2A enrichments at its promoter site (percentage bound around 36% than NC cells) (Fig. 5B). TGF-β1 luciferase reporter assay indicated that TRIM37 knockdown significantly reduced the transcriptional activity of TGF-β1, while overexpressing TRIM37 promoted its activity (Fig. 5C). Consistently, TGF-β1 mRNA level also altered relatively (Fig. 5D). We then picked two typical tumor suppressor genes PTEN, TP53 for further validation. TRIM37 silencing observably reduced ub-H2A occupancy at the promoter sites of PTEN and TP53, indicating the decreasing repression of transcription, while overexpressing TRIM37 showed opposite effects (Fig. 5E). Taken together, abnormal TRIM37 in RCC could ubiquitinate H2A and promote its enrichments at the promoter sites of putative tumor suppressors, thus releasing the occupy of ub-H2A at TGF-β1 promoters and activating its activity.

Our findings raise the possibility that H2A ubiquitination can involves into RCC tumor progression mediated by TRIM37. Thereafter, selectively H2A ubiquitination inhibitor PRT4165 was performed to validate this point. We firstly found that PRT4165 could notably inhibit H2A ubiquitination levels (Figure S4F), and it also inhibited the ub-H2A and TGF-β1 level in cell with OE-TRIM37 (Overexpression-TRIM37) or shTRIM37, with no change of TRIM37 itself, indicating that TRIM37 functioned at the upstream of H2A and TGF-β1 (Fig. 5F). Similar to TRIM37 ablation, PRT4165 could also increase the occupancy of ub-H2A at TGF-β1 promoter, suggesting that PRT4165 mimic the effect of TRIM37 suppression (Fig. 5G). Functional assays further proved the repressive role of PRT4165 in RCC cells malignancies with abnormal TRIM37 (Fig. 5H). These results suggested that TRIM37 oncogenic value in RCC mainly relies on a H2A ubiquitinated manner.

### TRIM37 promotes RCC tumorigenesis and metastatic potential in xenograft models

**Fig. 6:**
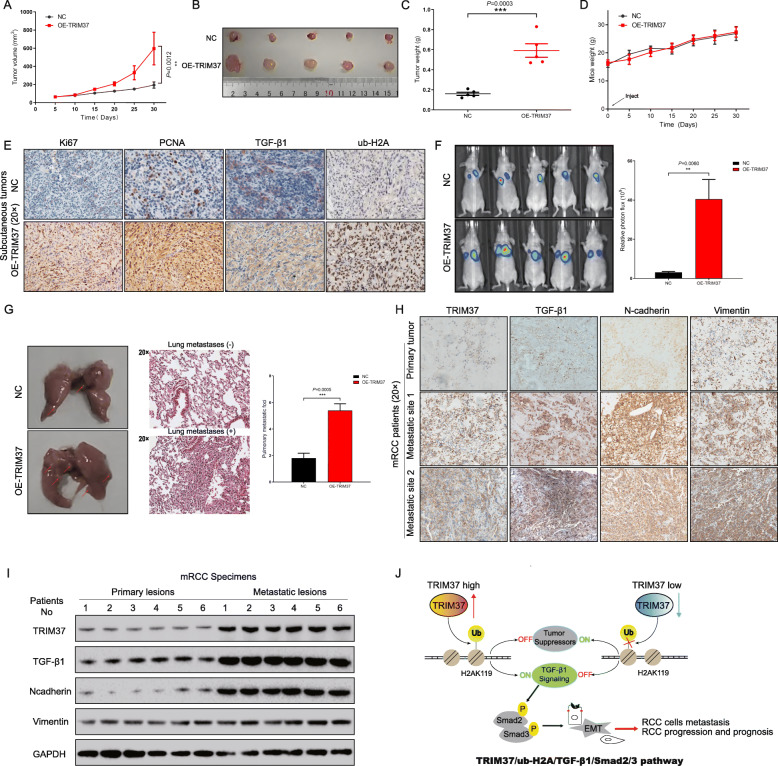
TRIM37 promotes RCC tumorigenesis and metastatic potential in xenograft models. **A,** TRIM37 overexpression significantly promoted the tumor volume of subcutaneous model than NC group. **B-C,** TRIM37 overexpression significantly improved the tumor size and weight than NC group**. D,** The mice weights gradually improved in both NC and TRIM37 overexpression group. There is no statistical difference between two groups. **E,** compared to NC group, tumors in TRIM37-overexpression group displayed significantly enhancive Ki67 and PCNA expression, as well as upregulated ub-H2A and TGF-β1 levels. **F,** Overexpression of TRIM37 accelerated more RCC cells to metastasize to pulmonary foci compared with control group, accompanied with higher fluorescence signal. **G,** More pulmonary foci were visibly observed in TRIM37 overexpression group. **H,** Using clinical RCC samples, TRIM37, TGF-β1 and EMT markers (N-cadherin and Vimentin) were correlatively upregulated in metastatic sites than primary lesions. **I,** Immunoblot assay demonstrated higher TRIM37, TGF-β1 and EMT markers in metastatic lesions than primary lesions. **J,** Model depicting the mechanism by which TRIM37 orchestrates RCC progression via TGF-β1/Smad2/3 signaling under a histone H2A ubiquitination-dependent manner

Given the essential role of TRIM37 in RCC progression, we further confirmed its tumorigenic function in vivo. We firstly constructed subcutaneous xenograft-transplanted tumor formation and tail vein injection metastatic models with nude mice. 786-O cells stably expressing TRIM37 were injected subcutaneously into nude mice. Concordant with in vitro both 2D and 3D findings, overexpressing TRIM37 of 786-O cells showed increased tumor growth rates (Fig. 6A-D). Compared to control group, tumor xenograft generated from TRIM37 overexpression cells displayed significantly enhancive Ki67 and PCNA expression, upregulation of ub-H2A and TGF-β1 levels were also observed (Fig. 6E). These results suggest that TRIM37 promote renal xenograft tumor growth in vivo. We next examined whether TRIM37 could modulate coincident metastatic and invasive phenotype in vivo. 786-O cells with stable TRIM37 expression were first labeled with luciferase system, and then injected into the tail veins of nude mice to construct metastatic organ models. Using a whole-body fluorescence system, we demonstrated that the luciferase signals intensity of OE-TRIM37 group were remarkably higher than those in NC group (Fig. 6F), of which mainly concentrating on pulmonary fields. More pulmonary metastatic foci were observed in OE-TRIM37 group when compared with those of NC group (Fig. 6G). H&E staining was further performed on the pulmonary tissue to validate the metastatic sites (Fig. 6G). Next, we verified the expression correlation with clinical samples from metastatic sites of RCC patients. TRIM37, TGF-β1 and relative EMT markers were correlatively upregulated in metastatic sites than primary tumors (Fig. 6H, I), indicating the high relevance of this aberrant signaling. Collectively, these results demonstrate that TRIM37 promotes RCC progressive phenotype in vivo.

## Discussion

Chromatin is composed of the nucleosome, which describes the complex of 147 base pairs of DNAs wrapped around a histone octamer [36]. Accumulating researches have revealed the covalent modification of histone code models in multiple DNA-based processes, including transcription, DNA repair and replication [37, 38]. Among which, histones ubiquitination has been reported in a series of cellular events. H2A, as a core member of histones, its ubiquitylation was highly mapped to Lys119 site and displayed abundant modification [39]. The role of H2A ubiquitination in transcriptional regulation and chromatin remodeling has been extensively studied. H2AK119 mono-ubiquitination was catalyzed by CRL4B, and further promoted cells tumorigenesis [40]. H2A was deubiquitinated by deubiquitinating enzyme USP16, and deubiquitinating of H2A is required for the activation of androgen receptor (AR)-mediated genes in prostate cancers [41]. TRIM proteins describe a category of E3 ubiquitin ligases as they consist of a common RING-finger domain. The role of TRIM family in polyubiquitination modifications has been explored among tumor diseases [42, 43]. Beyond this, the function of TRIM proteins in mono-ubiquitination especially for histone H2A has not been fully uncovered. In this study, we identified TRIM37 as a crucial E3 ubiquitin ligase that could directly accelerate H2A mono-ubiquitination and thereby activate TGF-β1/Smad2/3 signaling in RCC progression.

Rare is known about the role of TRIM37 in renal cancer. TRIM37 accounts for 5% alterations, among which 69% are mRNA high, implying the critical existence of high TRIM37 transcriptome in RCC. High TRIM37 score was associated with advanced tumor grade and poorer survival. These data affirmed TRIM37 as an oncogenic driven gene in RCC. Furthermore, TRIM37 significantly enhanced RCC cells invasive and metastatic potential in both vitro and vivo assays. Previous studies have shown that EMT as a critical procedure in tumorigenic progression, malignant migration, metastasis and resistance to therapy as well [44], however its role in RCC is not well characterized. We showed RCC cells with TRIM37 overexpression displayed mesenchymal phenotype, indicating a promoting role in epithelial to mesenchymal state transition.

EMT phenotypic process is usually driven by several essential transcriptional factors including SNAIL, ZEB-1 and TGF-β1. TGF-β1 is considered as a potent inducer in developing EMT process and cancer development [45]. Knocking down TRIM37 remarkably inhibited TGF-β1/Smad2/3 signaling, with decrease of transcriptional factors SNAIL and ZEB-1. As reported, oncogenic TGF-β1 signaling is associated with tumor development and EMT in cancer metastases [35, 45, 46], however its role in RCC is not fully elucidated. Based on TCGA datasets and our RCC cohort, the dominant mRNA-high alteration across TRIM37 and TGF-β1 signaling still dictated the clinical. However, we should notice that TGF-β1 signaling has a dichotomous role in tumorigenesis, acting as a tumor suppressor in early stages and tumor promoter in late stages [47]. In late tumor stages, TGF-β1 signaling activation induces Smad2/3 phosphorylation and translocation to nucleus, thus exerting tumor EMT process, invasiveness and metastasis. Our study mainly demonstrated the promoting role of TRIM37 in RCC EMT process and cell metastasis, potentially via TGF-β1/Smad2/3 signaling activation. Therefore, the TGF-β1 signaling might primarily exerts an oncogenic and pro-metastatic function in this condition. Taken together, we could preliminarily clarify that the promoting function of TRIM37 in RCC tumor progression relies on TGF-β1 signaling. However, considering the complexity of TRIM family function, we believe that additional regulatory mechanisms may also exist.

We next tried to explore the mechanistic link between TRIM37 and TGF-β1. Previous studies showed that TRIM37 exerted histone H2A ubiquitination and drive centrosome dysfunction of breast cancer [10, 48]. H2A ubiquitinating modification is an essential routine for transcriptional regulation, and this point put us forward whether TRIM37 could activate TGF-β1 signaling via H2A ubiquitinating way. Our results showed TRIM37 directly bound H2A and ub-H2A, and its occupancy at TGF-β1 promoter significantly decreased after TRIM37 gain of function. The decreased enrichments at TGF-β1 promoter could release its transcriptional ability, which is consistent with high expression of TGF-β1 after TRIM37-overexpression in our study. It’s well known that H2A ubiquitination causes transcriptional silencing, however our study shows its ubiquitination could activate oncogenic TGF-β1 signaling under certain condition. Interestingly, the ub-H2A enrichments at promoters of typical suppressors PTEN and TP53 significantly reduced when TRIM37 was knocked down, indicating a transcriptional activation of tumor suppressors. Therefore, this could be a probable explanation that TRIM37 promote ub-H2A enrichments at the promoters of tumor suppressors, resulting in a reduce of TGF-β1 promoter occupancy and signaling activation. However, we didn’t illustrate how ub-H2A was transferred between different gene promoters, especially between tumor suppressors and oncogenic factors. Other ubiquitination enzymes like E1 and E2 might exert specific function in the regulation, which still needs further investigation. PRT4165 is a well-established H2A ubiquitylation inhibitor and has been verified to involve into cancer targeted therapies [49], suppression of malignant cells [50], as well as ubiquitylation inhibition at DNA double-strand breaks [51]. We showed that PRT4165 as a specific ubH2A inhibitor could markedly blockade the promoting H2A-ubiquitination state in TRIM37 overexpression RCC cells. PRT4165 also significantly increased the ub-H2A occupancy at TGF-β1 promoter sites, which is similar to TRIM37 knockdown effect. Additionally, decreased cell invasive viability with restrained TGF-β1 signaling were also observed upon PRT4165 treatment, suggesting that the tumorigenic function of TRIM37/TGF-β1 signaling in RCC mainly relies on H2A ubiquitinating modification.

## Conclusions

Our study firstly identified the oncogenic and predictive role of TRIM37 in renal cell carcinoma. Through mechanistically studies, we constructed a novel regulating axis of TRIM37/ub-H2A/TGF-β1/Smad2/3 signaling in regulating RCC tumor metastases and progression (Fig. 6J). These findings will broaden the perspective of TRIM37 in urological neoplasms and it’s of great value to develop therapeutic approaches targeting TRIM37 ubiquitination route for potential interventions.

## Supplementary Information

**Additional file 1: Figure S1.** TRIM37 alteration in pRCC and its prognostic role. **A,** Heat map of altered genes in microarray chip of RCC. **B,** In RCC patients of NMU-Cohort 1, upregulating TRIM37 was correlated with advanced tumor grader but not stage statistically. C, Alterations between TRIM37 and putative RCC driven genes in TCGA datasets. TRIM37 had 5% alteration, and others PIK3CA (8%), MTOR (12%), PTEN (8%) and TP53 (9%). **D,** TRIM37 had 11% alteration in pRCC patients, as compared with other putative driven genes. **E,** Tumors with TRIM37 gain or amplification had higher mRNA levels than shadow deletion or diploid alterations in pRCC cohort.

**Additional file 2: Figure S2.** The promoting role of TRIM37 in RCC cells migration, invasion and proliferation. **A,** Construction and validation of knocking down or overexpressing TRIM37 cell models (Caki-2, ACHN). **B,** Knockdown of TRIM37 attenuated migration and Matrigel invasion ability of Caki-2 cells. **C,** Knockdown of TRIM37 inhibited scratch healing ability of Caki-2 cells. **D,** Overexpressing TRIM37 promoted cell migration and Matrigel invasion ability in ACHN lines. **E,** Overexpression of TRIM37 enhanced scratch healing ability of ACHN cells. **F,** Knockdown of TRIM37 attenuated cell colony formation ability in Caki-1 and Caki-2 cells. G, Knockdown of TRIM37 inhibited cell proliferation ability in Caki-1 and Caki-2 cells. **H-I,** Overexpression of TRIM37 showed limited promoting role in RCC cells colony formation and growth in 786-O and ACHN lines. **J,** Models illustrating EMT program patterns, indicating a loss of epithelial phenotypes and gain of mesenchymal characteristics. **K,** TRIM37 influenced the expression of EMT markers in Caki-2 and ACHN lines.

**Additional file 3: Figure S3.** Alterations of TRIM37, TGF-β1 signaling and EMT markers in TCGA ccRCC or pRCC cohorts, and TGF-β1’s role in RCC prognosis. **A,** Alterations of TRIM37, TGF-β1 signaling and EMT markers in TCGA ccRCC or pRCC cohorts. In ccRCC cohort, TRIM37 accounts for 5% alteration, TGF-β1 4%, SMAD2 7%, SMAD3 4%, SNAIL1 4%, ZEB1 7%, CHD1 4%, CHD2 8% and VIM 7%. In pRCC cohort, TRIM37 alteration rates 11%, TGF-β1 5%, SMAD2 8%, SMAD3 5%, SNAIL1 1.1%, ZEB1 5%, CHD1 5%, CHD2 2.5% and VIM 7%. **B,** In GEO dataset (GSE11151), TGF-β1 was significantly upregulated in RCC tumors than normal tissues. C, High expression of TGF-β1 was associated with shorter overall survival period in RCC patients of TCGA dataset.

**Additional file 4: Figure S4.** Go and KEGG analyses of TRIM37 connected gene signatures, and TRIM37 affects ub-H2A levels. **A**, A total of 26 genes were identified to interact with TRIM37 base on PPI network analysis in STRING datasets. **B**, GO biological process analysis recruited several significant enrichments, including “regulation of cellular macromolecule biosynthetic process”, “regulation of primary metabolic process”, et al. **C**, GO cellular component analysis identified that “CD40 receptor complex”, “intracellular-bounded organelle” and “PcG protein complex” was enriched. **D**, KEGG pathway enrichments identified several markedly pathways, including “small cell lung cancer”, “NF-kappa B signaling pathway”, “IL-17 signaling pathways”, et al. **E**, Overlapping gene sets of TRIM37 and ub-H2A chip-chip data from GSE48196: a total of 7638 genes were overlapped in both TRIM37 and ub-H2A chip-chip data. **F**, Specific ub-H2A inhibitor PRT4165 significantly abolished ubiquitinating modifications in Caki-1 and 786-O cells.

**Additional file 5: Table S1**: Clinical baseline of patients in NMU_RCC cohort 1.

**Additional file 6: Table S2** Clinical characteristics of RCC patients in TMAs.

**Additional file 7.**

## Data Availability

The analyzed datasets in the study are available from public datasets or the urology research lab on reasonable request.

## Abbreviations

RCC: Renal cell carcinoma
TRIM: Tripartite motifs
TMA: Tissue microarray
shRNA: small hairpin RNA
NC: Negative control
EMT: Epithelial-mesenchymal transition
IF: Immunofluorescence
IHC: Immunohistochemistry
Phos-Smad2: Phosphorylation-Smad2
Phos-Smad3: Phosphorylation-Smad3
