# Supplementary figures and images for: TRIM37 orchestrates renal cell carcinoma progression via histone H2A ubiquitination-dependent manner
Source: J Exp Clin Cancer Res. 2021 Jun 15;40:195. doi: 10.1186/s13046-021-01980-0 (PMC8204444; doi:10.1186/s13046-021-01980-0)

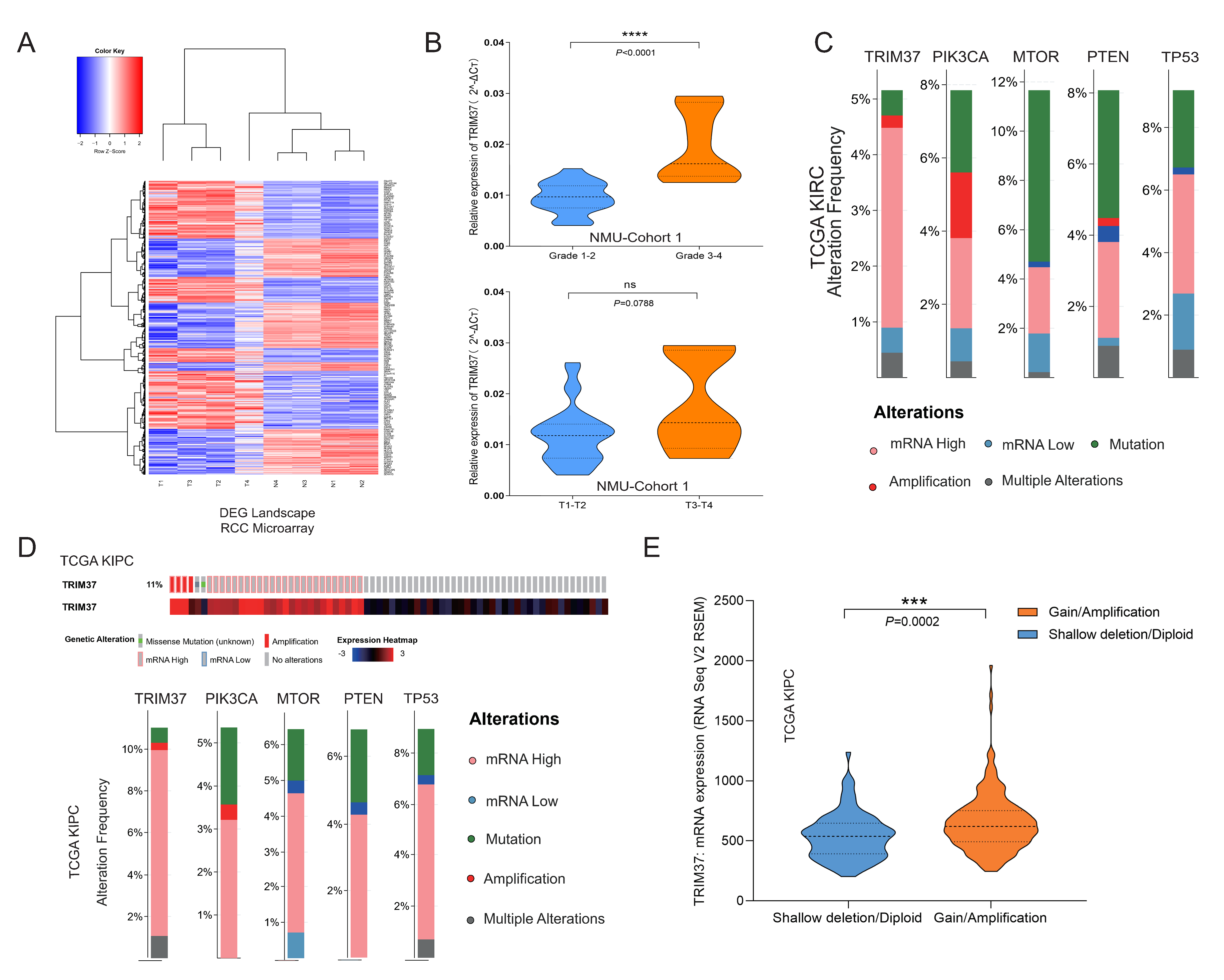

Supplement: Supplementary file 1 — Additional file 1: Figure S1. TRIM37 alteration in pRCC and its prognostic role. A, Heat map of altered genes in microarray chip of RCC. B, In RCC patients of NMU-Cohort 1, upregulating TRIM37 was correlated with advanced tumor grader but not stage statistically. C, Alterations between TRIM37 and putative RCC driven genes in TCGA datasets. TRIM37 had 5% alteration, and others PIK3CA (8%), MTOR (12%), PTEN (8%) and TP53 (9%). D, TRIM37 had 11% alteration in pRCC patients, as compared with other putative driven genes. E, Tumors with TRIM37 gain or amplification had higher mRNA levels than shadow deletion or diploid alterations in pRCC cohort. [file 13046_2021_1980_MOESM1_ESM.tif]

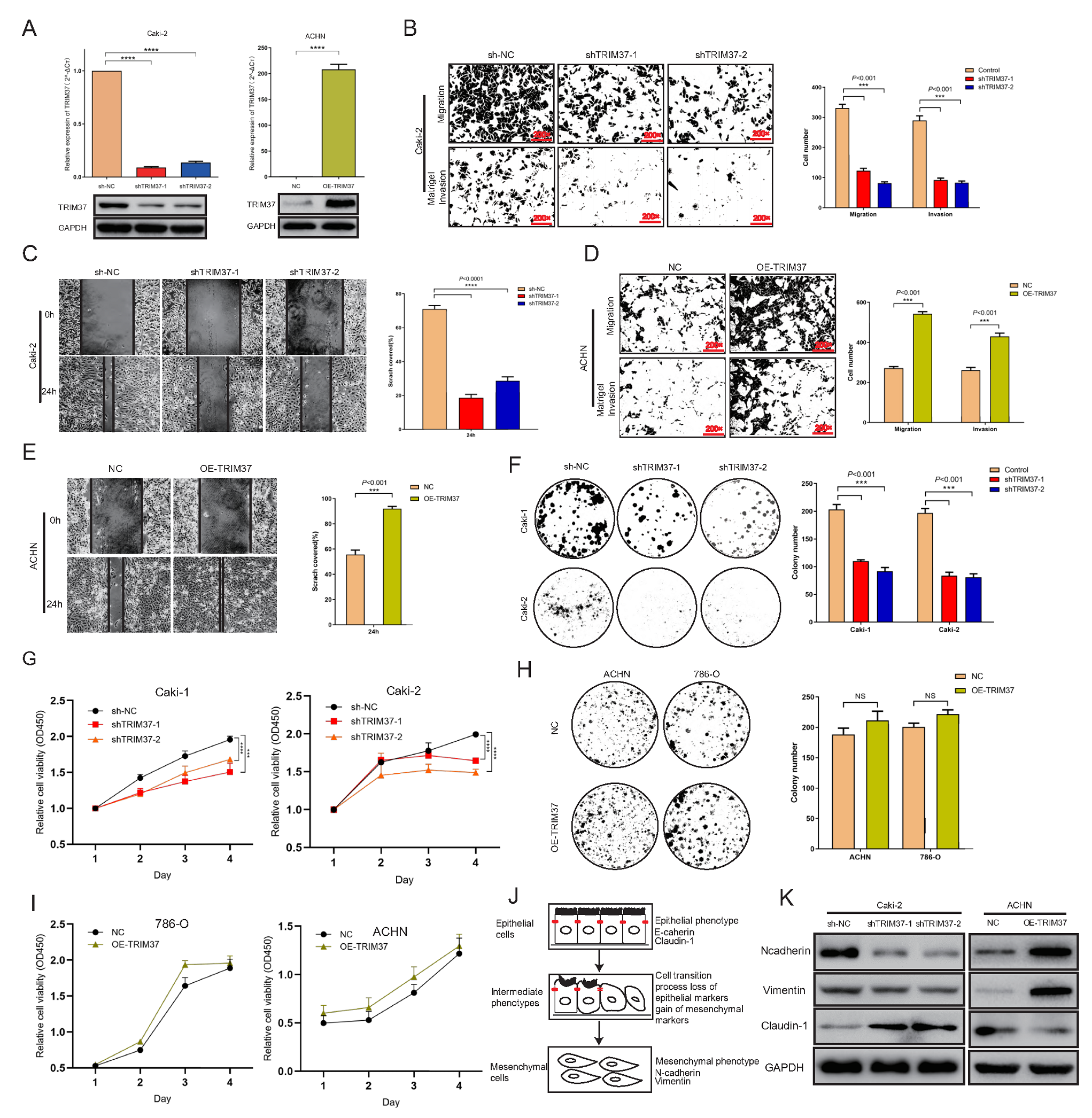

Supplement: Supplementary file 2 — Additional file 2: Figure S2. The promoting role of TRIM37 in RCC cells migration, invasion and proliferation. A, Construction and validation of knocking down or overexpressing TRIM37 cell models (Caki-2, ACHN). B, Knockdown of TRIM37 attenuated migration and Matrigel invasion ability of Caki-2 cells. C, Knockdown of TRIM37 inhibited scratch healing ability of Caki-2 cells. D, Overexpressing TRIM37 promoted cell migration and Matrigel invasion ability in ACHN lines. E, Overexpression of TRIM37 enhanced scratch healing ability of ACHN cells. F, Knockdown of TRIM37 attenuated cell colony formation ability in Caki-1 and Caki-2 cells. G, Knockdown of TRIM37 inhibited cell proliferation ability in Caki-1 and Caki-2 cells. H-I, Overexpression of TRIM37 showed limited promoting role in RCC cells colony formation and growth in 786-O and ACHN lines. J, Models illustrating EMT program patterns, indicating a loss of epithelial phenotypes and gain of mesenchymal characteristics. K, TRIM37 influenced the expression of EMT markers in Caki-2 and ACHN lines. [file 13046_2021_1980_MOESM2_ESM.tif]

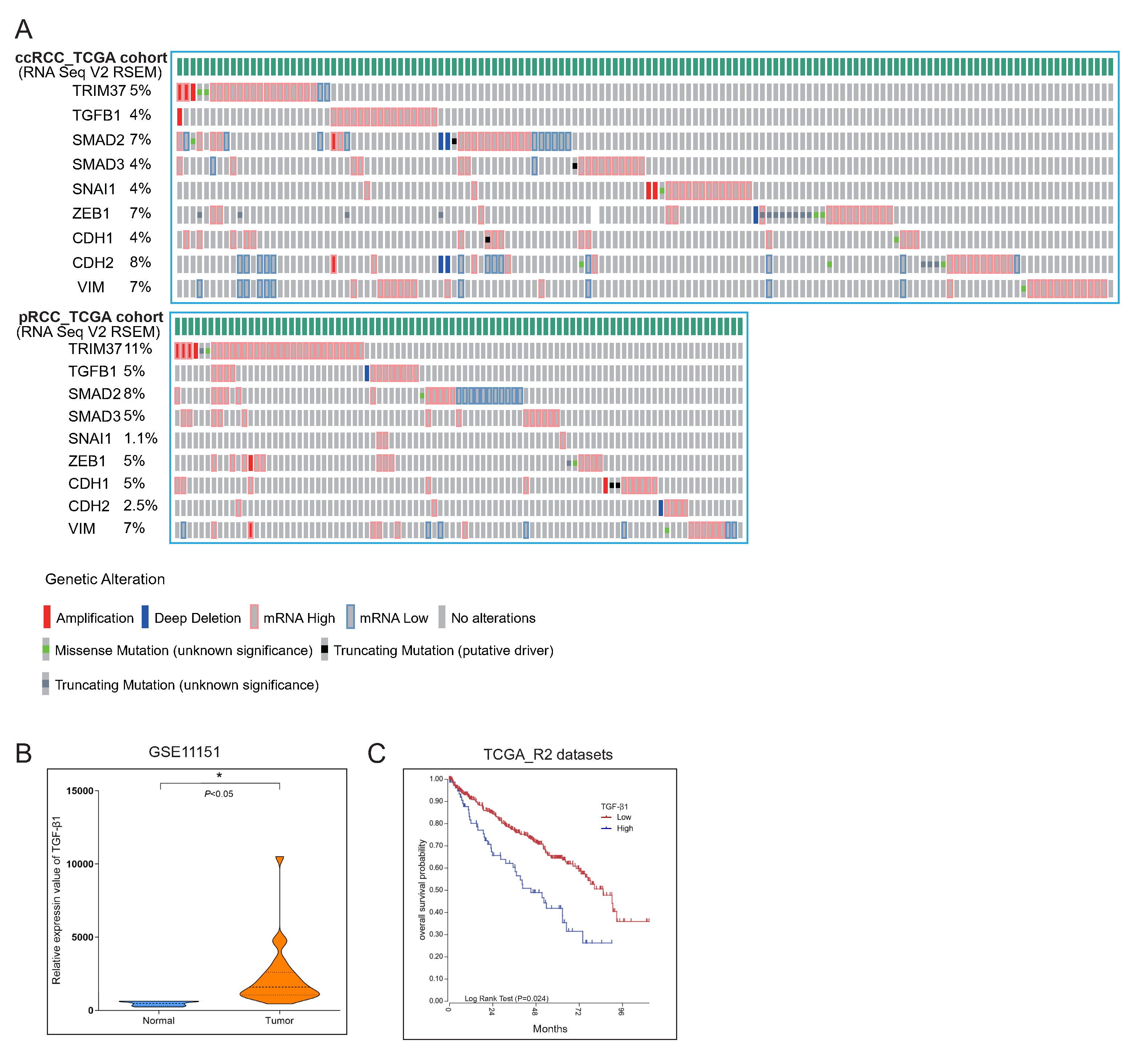

Supplement: Supplementary file 3 — Additional file 3: Figure S3. Alterations of TRIM37, TGF-β1 signaling and EMT markers in TCGA ccRCC or pRCC cohorts, and TGF-β1’s role in RCC prognosis. A, Alterations of TRIM37, TGF-β1 signaling and EMT markers in TCGA ccRCC or pRCC cohorts. In ccRCC cohort, TRIM37 accounts for 5% alteration, TGF-β1 4%, SMAD2 7%, SMAD3 4%, SNAIL1 4%, ZEB1 7%, CHD1 4%, CHD2 8% and VIM 7%. In pRCC cohort, TRIM37 alteration rates 11%, TGF-β1 5%, SMAD2 8%, SMAD3 5%, SNAIL1 1.1%, ZEB1 5%, CHD1 5%, CHD2 2.5% and VIM 7%. B, In GEO dataset (GSE11151), TGF-β1 was significantly upregulated in RCC tumors than normal tissues. C, High expression of TGF-β1 was associated with shorter overall survival period in RCC patients of TCGA dataset. [file 13046_2021_1980_MOESM3_ESM.tif]

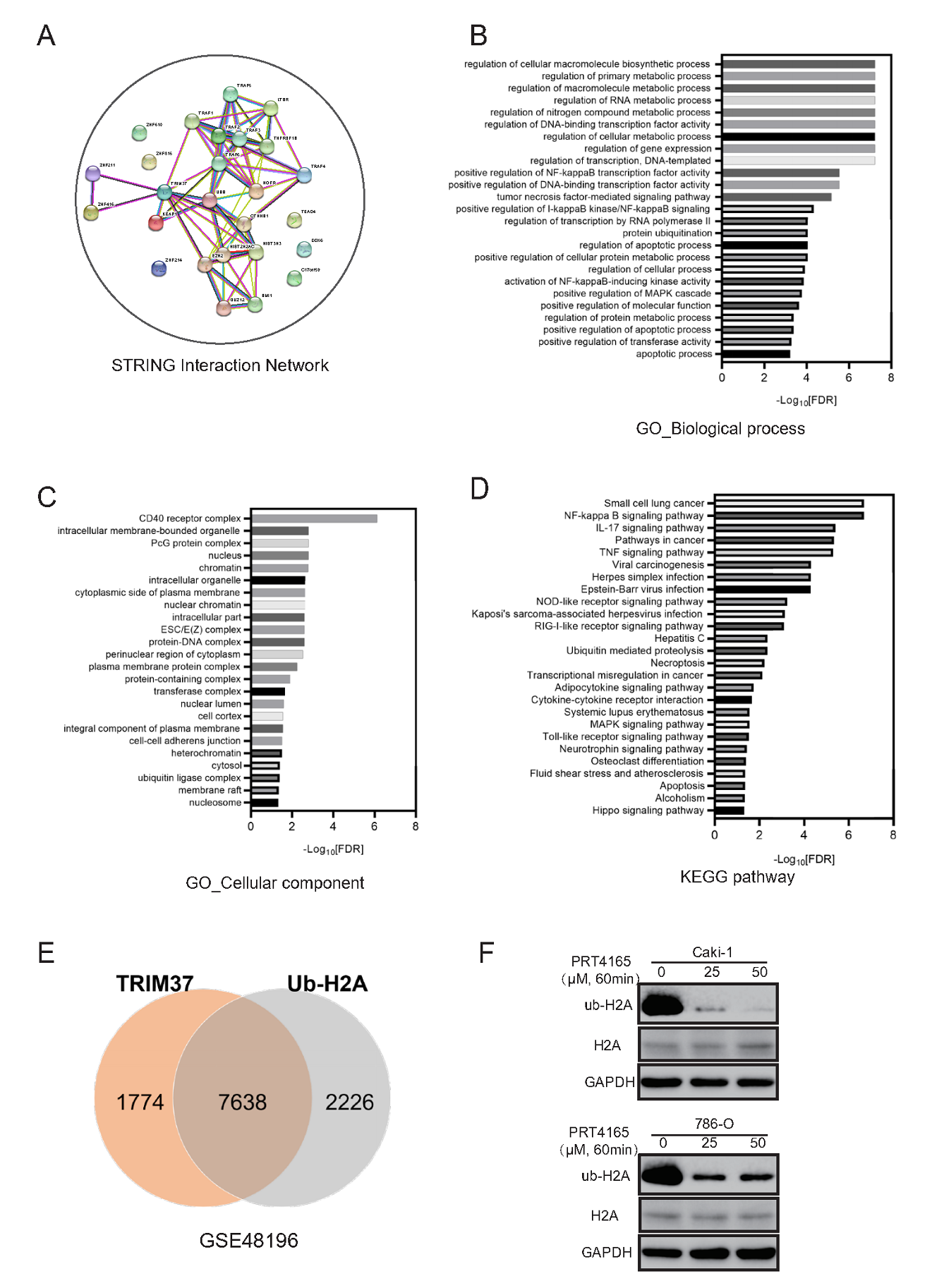

Supplement: Supplementary file 4 — Additional file 4: Figure S4. Go and KEGG analyses of TRIM37 connected gene signatures, and TRIM37 affects ub-H2A levels. A, A total of 26 genes were identified to interact with TRIM37 base on PPI network analysis in STRING datasets. B, GO biological process analysis recruited several significant enrichments, including “regulation of cellular macromolecule biosynthetic process”, “regulation of primary metabolic process”, et al. C, GO cellular component analysis identified that “CD40 receptor complex”, “intracellular-bounded organelle” and “PcG protein complex” was enriched. D, KEGG pathway enrichments identified several markedly pathways, including “small cell lung cancer”, “NF-kappa B signaling pathway”, “IL-17 signaling pathways”, et al. E, Overlapping gene sets of TRIM37 and ub-H2A chip-chip data from GSE48196: a total of 7638 genes were overlapped in both TRIM37 and ub-H2A chip-chip data. F, Specific ub-H2A inhibitor PRT4165 significantly abolished ubiquitinating modifications in Caki-1 and 786-O cells. [file 13046_2021_1980_MOESM4_ESM.tif]
