# Supplementary material for: TRIM37 orchestrates renal cell carcinoma progression via histone H2A ubiquitination-dependent manner
Source: J Exp Clin Cancer Res. 2021 Jun 15;40:195. doi: 10.1186/s13046-021-01980-0 (PMC8204444; doi:10.1186/s13046-021-01980-0)
Supplement: Supplementary file 5 — Additional file 5: Table S1: Clinical baseline of patients in NMU_RCC cohort 1. [file 13046_2021_1980_MOESM5_ESM.docx]

| Age |  |
| --- | --- |
| Mean±SD, year | 56.11±11.23 |
| <60 | 9 |
| ≥60 | 38 |
| Gender |  |
| Male | 30 |
| Female | 17 |
| Histological subtype |  |
| Clear cell RCC | 38 |
| Papillary RCC | 3 |
| Others | 6 |
| Histologic grade |  |
| Ⅰ-Ⅱ | 25 |
| Ⅲ-Ⅳ | 13 |
| Others | 9 |
| T stage |  |
| T1-T2 | 36 |
| T3-T4 | 11 |

Table S1: Clinical baseline of patients in NMU_RCC cohort 1
