# Supplementary material for: TRIM37 orchestrates renal cell carcinoma progression via histone H2A ubiquitination-dependent manner
Source: J Exp Clin Cancer Res. 2021 Jun 15;40:195. doi: 10.1186/s13046-021-01980-0 (PMC8204444; doi:10.1186/s13046-021-01980-0)
Supplement: Supplementary file 6 — Additional file 6: Table S2 Clinical characteristics of RCC patients in TMAs. [file 13046_2021_1980_MOESM6_ESM.docx]

Table S2 Clinical characteristics of RCC patients in TMAs

| Age |  |  |
| --- | --- | --- |
| Mean±SD, year | 56.34±13.72 |  |
| ＜60 | 80 | 60.15% |
| ≥60 | 53 | 39.85% |
| Gender |  |  |
| Male | 83 | 62.41% |
| Female | 50 | 37.59% |
| Tumor size |  |  |
| ≤4 | 71 | 53.38% |
| >4 | 62 | 46.62% |
| Histological subtype |  |  |
| Clear cell RCC | 118 | 88.72% |
| Papillary RCC | 6 | 4.51% |
| Others | 9 | 6.77% |
| Histologic grade |  |  |
| Ⅰ | 28 | 21.05% |
| Ⅱ | 82 | 61.65% |
| Ⅲ | 19 | 14.29% |
| Ⅳ | 4 | 3.01% |
| T stage |  |  |
| T1 | 115 | 86.47% |
| T2-T4 | 18 | 13.53% |
| Death |  |  |
| Yes | 18 | 13.53% |
| No | 115 | 86.47% |
| Recurrence |  |  |
| Yes | 12 | 9.84% |
| No | 110 | 90.16% |
| TRIM37 expression |  |  |
| Low | 79 | 59.40% |
| High | 54 | 40.60% |
